# Editorial: Women in psychiatry 2024: computational psychiatry

**DOI:** 10.3389/fpsyt.2025.1692575

**Published:** 2025-09-26

**Authors:** Chen Zhang, Andreea Oliviana Diaconescu, Xi Zhu

**Affiliations:** ^1^ Department of Bioengineering, University of Texas at Arlington, Arlington, TX, United States; ^2^ Krembil Centre for Neuroinformatics, Centre for Addiction and Mental Health, Toronto, ON, Canada; ^3^ Department of Psychiatry, University of Toronto, Toronto, ON, Canada

**Keywords:** women in computational psychiatry, psychotic-like experience (PLE), belief updating, HOIRT, bipolar disorder, patient readmission, socially interactive agents

As part of ongoing efforts to address and achieve representation of women in research, the Frontiers Research Topic series, “*Women in Psychiatry: Computational Psychiatry*” was launched in 2023. In the past 3 years, gender disparities have been improving within natural sciences, characterized by 41% of female authorship in 2024 (1). The gender disparities in science vary across fields, from 53% in pediatric medicine to 15% within classical physics. In 2018, 16% of corresponding authors are women, and in 2024, 17% identified as women, demonstrating an increase of 1% (2, 3). The gender disparity has been improving at a slower rate. In addition, gender disparity is also present and varies across countries; the United States, France, and Canada had the highest women scientist ratio of 34%, whereas the United Kingdom came at 29%, and Japan at 16%. These data suggests that gender disparities persist in 2025.

The Women in Psychiatry series aims to promote equity, diversity, and inclusion in computational psychiatry by supporting women scientists. This 2024 editorial features four peer-reviewed articles from authors affiliated with Asian, Middle Eastern, European, South American, and North American universities. Of these contributions, 100% were handled by female editors. In terms of authorship, 62% of all authors (15 over 24), 100% of corresponding authors, and 100% of lead authors were women. This higher representation of leading women authors promotes and increases the visibility of female colleagues, and we are proud to feature four diverse contributions to the field. Studies were performed across different populations, ranging from healthy individuals to psychiatric populations with bipolar disorder (BD), in a diverse research design varying from observational, descriptive, and correlational using different computational modeling methods.

Different computational methods were used to understand the computational mechanism underlying psychological and cognitive processes. Fromm et al. employed a normative learning model to examine the relationship between belief uncertainty and psychotic-like experience (PLE) within sub-clinical populations. They found that higher PLE is associated with slower belief updating in scenarios of large prediction error. This suggests that within conditions characterized by delusion-like rigid beliefs, there is a resistance to override and update the previously existing beliefs. Future study should build on this and examine whether belief acquisition, the process through which one acquires a new belief, is also equally impacted within PLE.

Yao et al. studied early detection of cognitive impairment (CI) in 86 patients recruited from primary care through employing diverse machine learning models. CI was determined based on previous diagnosis, medical record, or assessments completed within the last 18 months. They generated composite scores using the High Order Item Response Theory (HOIRT) model based on the Dimensional Change Card Sort (DCCS), Picture Sequence Memory (PSM), Flanker, Working Memory (MFS), and Number-Symbol Mismatch (NM) assessments, along with demographic features such as age. Among the machine learning approaches. The Random Forest classifier using HOIRT-derived composition score produced the best classification performance. This study illustrates that relying on a single, simple cut point for a composite score is insufficient to predict cognitive impairment, and the HOIRT generated composite score can be a valuable tool for predicting impairment in clinical settings.

Palacios-Ariza et al. conducted three ML models: decision trees, random forest, and support vector machine to predict patient admission and readmissions in 2725 patients with bipolar disorder using electronic health records. Recursive feature elimination and sequential feature selection were implemented to reduce standard error and dimensionality of the 21 candidate predictors. Random forest achieved the highest performing accuracy in predicting admissions (AUC = 0.98), while recursive feature elimination identified the number psychiatric emergency visits as the candidate predictor with the greatest predictive power for admissions. Random Forest remained the best performing model in predicting the readmissions within bipolar patients, but reporting weaker predictive accuracy of AUC below 0.70. This work highlights the potential of utilizing EHR data to compensate for the scarcity of data to achieve comprehensive mental health care, individualized treatment, monitor of individual mental health progression, thereby achieving clinical translation.

These three studies highlight the strength of computational methods in investigating and revealing the cognitive mechanism underlying psychological conditions. The computational method was not only limited to investigating the basic science, but machine learning methods were also used for clinical prediction and application within bipolar disorder. In addition, the fourth study proposes the development of a toolkit that aids the investigation of working therapeutic alliance, an important variable predictive of patient therapeutic outcome, between client and clinicians. Movement synchrony, defined as the: “temporal coordination of gesture and postures between interaction partners”, consists of five criteria: context, modality, resources, entrainment, and time lags. Movement synchrony reflects inter-brain coupling of two interactional partners and enhances relational alliance within two interactional partners. Research has found positive association between movement synchrony, therapeutic alliance, and treatment outcome. However, movement synchrony has been challenging to investigate due to obstacles in manipulating and controlling for synchronous behavior within human psychotherapeutic settings. Therefore, Wessler et al. proposed the development of an experimental toolkit with socially interactive agents (SIA) with human-controlled ability, with the following technological components such as social signal analysis, automatic speech recognition, natural language understanding, and large language models. The SIA is constructed with controlled parameters that allows the SIA to respond to detect social signals with time delays, movement intensity, and movement modality (head, body, other combinations). The SIA allows investigation of research questions, including but not limited to, relationships of therapeutic alliance, movement synchrony, and attachment styles. SIA bridges the intersection of clinical application, understanding treatment efficacy, and enhancing treatment efficacy.

Collectively, this editorial has compiled a series of psychiatry research that advances in theory, experimental design, and methodology. The diversity of research is not only limited within the design, but as well as the authors’ cultural background. Acknowledging and promoting the equity, diversity, and inclusivity of research will enable a diversity of voices and creativity. We hope this series of research serves as inspiration for readers to celebrate, reflect, understand, and further create and contribute to a culturally inclusive, innovative, and collaborative research environment for all scientists.
